# Expression of Concern: Seroepidemiology and associated risk factors of hepatitis B and C virus infections among pregnant women attending maternity wards at two hospitals in Swabi, Khyber Pakhtunkhwa, Pakistan

**DOI:** 10.1371/journal.pone.0345147

**Published:** 2026-03-19

**Authors:** The PLOS One Editors

The *PLOS One* Editors issue an Expression of Concern to alert readers that this article [1] was identified as one of a series of submissions for which we have concerns about adherence to the journal’s authorship policy. We regret that these issues were not addressed prior to the article’s publication.
