# Supplementary material for: Seroepidemiology and associated risk factors of hepatitis B and C virus infections among pregnant women attending maternity wards at two hospitals in Swabi, Khyber Pakhtunkhwa, Pakistan
Source: PLoS One. 2021 Aug 20;16(8):e0255189. doi: 10.1371/journal.pone.0255189 (PMC8384242; doi:10.1371/journal.pone.0255189)
Supplement: S1 File — (DOCX) [file pone.0255189.s001.docx]

The following document is the details of tools or questionnaires used in this research work for collecting information/data on socio-demographic characteristics background, possible risk factors and laboratory investigation report for each study participant (***Supplementary file for this work***).

**Organizations**: - Department of Biology, University of Haripur, Departments of Gynecology and Obstetrics Bacha Khan Medical Complex Shahmansoor and District Head Quarter Hospital Swabi.

**Title of the study**: - Seroepidemiology and Associated Risk Factors of Hepatitis B and C Virus Infections among Pregnant Women Attending Maternity Wards at Two Hospitals in Swabi, Khyber Pakhtunkhwa, Pakistan which is the major cause of mortality and morbidity, and for provision of recommendation for possible prevention and controlling the problem and help them for effective intervention plan in the future.

The involvement in this study is based on your voluntary and you have the right to refuse to participate in the study, and the confidentiality of the information gathered will be kept secret and only used for this study. The result of the laboratory finding will be communicated to your physician or care giver.

**Direction:-**Please encircle the letter of your answer or correctly fill in the blank space provided for open ended questioners.

## Part-I: Socio demography survey questioner

1. Identification:

Date: _________________ Code No: ______________

Address: ______________ Age (Years): __________________

1. Where do you live? A. Urban B. Rural
2. What is your educational status?
3. Illiterate B. Primary
4. C. Secondary D. Above (Higher education)
5. What is your occupation?
6. Farmer B. Housewife C. Private
7. Daily laborer E. Governmental Employee F. NGO employee

F-Other, specify ____________________

1. What is your marital status?
2. Married B. Unmarried
3. What is your family monthly income (specify) ________________________
4. What is the number of your family members________________________
5. What is the number of your children alive________________________
6. Parity A. primigravidae B. Multigravidae, C. Grand multipara

**Part II: Questions related to HBV and HCV risk factors**

1. Have you ever practiced the following?
2. Ear/Nose piercing (in jeweler’s shop) A. yes B. No
3. Tattooing on body A. yes B. No
4. Dental extraction at home A. yes B. No
5. Dental extraction at health facility A. yes B. No
6. Shaving eyebrow A. yes B. No
7. Ex delivery at Health facility A. yes B. No
8. Abortion A. yes B. No
9. Hospital admission A. yes B. No

If yes why? ________

1. Surgical procedure A. yes B. No
2. Receiving blood transfusion A. yes B. No
3. History of Contact with jaundiced Patient A. yes B. No
4. Venous or body piercing for treatment A. yes B. No
5. History of Sexually transmitted disease (STD) A. yes B. No
6. No risk A. yes B. No
7. Have Laboratory tests been done?

|  |  | 1. Yes 2. No | If yes please list below |
| --- | --- | --- | --- |
|  |  | Base line | Current status |
| 1. |  |  |  |
| 2. |  |  |  |
| 3. |  |  |  |
| 4. |  |  |  |
| 5. |  |  |  |

| Test type | HBsAg test result | | Anti-HCV test result | |
| --- | --- | --- | --- | --- |
| Laboratory test result | +Ve | -Ve | +Ve | -Ve |
| ICT |  |  |  |  |
| ELISA |  |  |  |  |

1. Type of laboratory test

We thank you for your response!
