# Supplementary material for: Seroepidemiology and associated risk factors of hepatitis B and C virus infections among pregnant women attending maternity wards at two hospitals in Swabi, Khyber Pakhtunkhwa, Pakistan
Source: PLoS One. 2021 Aug 20;16(8):e0255189. doi: 10.1371/journal.pone.0255189 (PMC8384242; doi:10.1371/journal.pone.0255189)
Supplement: Attachment — Submitted filename: reviewer comment April 18 2021.docx [file pone.0255189.s002.docx]

Title: Seroepidemiology and Associated Risk Factors of Hepatitis B and C Virus Infections among Pregnant Women Attending Maternity Wards at Two Hospitals in Swabi, Khyber Pakhtunkhwa, Pakistan

*Muhammad Israr1, 2*, Fawad Ali3, Arif Nawaz3, Muhammad Idrees4, Bashir Ahmad1, Rashid*

*5 Iqbal5, 2020*

**In the abstract:** there is no result conclusion indicated!

**Method part**

- Source population is not stated under method part
- Under method part: hierarchal permission process for the research was not described well and ethical principle was not stated well! (e.g. for those positive cases how the investigator manage the issue and also whether verbal or written consent was taken)
- Sampling techniques and sample size were not described.
- Quality assurance measures were not stated.
- Hospital and health center were used interchangeably (but not the same)
- Interpretation of the laboratory test for both viruses was not stated!
- Data analysis steps were not clear to understand how it was performed

**Result**

- Result part was not elaborated well (e.g by describing the socio-demographic factors except in the table)
- Prevalence of HBV and HCV, the socio-demographic factors and associated factors shall be stated separately under different subtitles as the objective
- In addition to table, if graphs are used to show the result, it will be good to easily understand the result in %.
- There must be an explanation for why “Dental extraction, Receiving blood transfusion, Educational level and Residential status associated with HBVsAg positivity and Surgical procedure, maternal age for HCV Ab positivity”

**Under discussion:**

- Strength and Limitation of the study shall be specified

**Under Conclusion**

- The result was not interpreted well whether it was high or low according to WHO guidelines for HBV and HCV (whether it falls in the endemic interval or hyper endemic interval and only risk factors were stated in line with the result.
