# Supplementary material for: Seroepidemiology and associated risk factors of hepatitis B and C virus infections among pregnant women attending maternity wards at two hospitals in Swabi, Khyber Pakhtunkhwa, Pakistan
Source: PLoS One. 2021 Aug 20;16(8):e0255189. doi: 10.1371/journal.pone.0255189 (PMC8384242; doi:10.1371/journal.pone.0255189)
Supplement: Attachment — Submitted filename: Responses to Editor and Reviwers.docx [file pone.0255189.s003.docx]

Dear Dr. Isabelle Chemin,

We are grateful to you and the reviewers for reading and constructive suggestions which improved our manuscript and for the opportunity to revise and resubmit. We are pleased to submit the revised manuscript entitled “Seroepidemiology and Associated Risk Factors of Hepatitis B and C Virus Infections among Pregnant Women Attending Maternity Wards at Two Hospitals in Swabi, Khyber Pakhtunkhwa, Pakistan” with Id PONE-D-21-11040 for consideration in PLOS ONE. On the following pages, you will find our response to the editor and reviewer comments. The editor and reviewer comments are highlighted with red color and responses to each point with bold text. On behalf of my co-authors, I thank you for your consideration of this resubmission. We appreciate your time and look forward to your response.

Sincerely,

Dr. Muhammad Israr, PhD, Postdoc (Corresponding Author)

Associate Professor, Department of Biology

The University of Haripur, KPK, Pakistan

Responses to Editor Comments:

Thank you for submitting your manuscript to PLOS ONE. After careful consideration, we feel that it has merit but does not fully meet PLOS ONE’s publication criteria as it currently stands. Therefore, we invite you to submit a revised version of the manuscript that addresses the points raised during the review process. More details are required in the results sections (see review); precisions in the Material and methods, discussion may be strengthened.

Please submit your revised manuscript by 2 months. If you will need more time than this to complete your revisions, please reply to this message or contact the journal office at plosone@plos.org. When you're ready to submit your revision, log on to https://www.editorialmanager.com/pone/ and select the 'Submissions Needing Revision' folder to locate your manuscript file.

**We are grateful to the editor for these constructive suggestions. The results, material and method and discussion sections were revised according to editor suggestions and highlighted with red color.**

Please include the following items when submitting your revised manuscript:

- A rebuttal letter that responds to each point raised by the academic editor and reviewer(s). You should upload this letter as a separate file labeled 'Response to Reviewers'
- A marked-up copy of your manuscript that highlights changes made to the original version. You should upload this as a separate file labeled 'Revised Manuscript with Track Changes'
- An unmarked version of your revised paper without tracked changes. You should upload this as a separate file labeled 'Manuscript'.

**A letter “Response to Reviewers” Revised Manuscript with Track Changes and Revised Manuscript without Track Changes are attached according to editor suggestions.**

1. Please ensure that your manuscript meets PLOS ONE's style requirements, including those for file naming. The PLOS ONE style templates can be found at

https://journals.plos.org/plosone/s/file?id=wjVg/PLOSOne_formatting_sample_main_body.pdf and

<https://journals.plos.org/plosone/s/file?id=ba62/PLOSOne_formatting_sample_title_authors_affiliations.pdf>

**The whole manuscript is revised according to the journal PLOS ONE style requirements**

2. PLOS requires an ORCID iD for the corresponding author in Editorial Manager on papers submitted after December 6th, 2016. Please ensure that you have an ORCID iD and that it is validated in Editorial Manager. To do this, go to ‘Update my Information’ (in the upper left-hand corner of the main menu), and click on the Fetch/Validate link next to the ORCID field. This will take you to the ORCID site and allow you to create a new iD or authenticate a pre-existing iD in Editorial Manager. Please see the following video for instructions on linking an ORCID iD to your Editorial Manager account:

<https://www.youtube.com/watch?v=_xcclfuvtxQ>

**The registered ORCID ID of the corresponding author is validated in the Editorial Manager.**

3. Thank you for including your ethics statement: "The ethical endorsement for study conduction was approved by the Institutional Research Ethical Committee (IREC) of BKMC and DHQ Hospital Swabi."

a) Please provide additional details regarding participant consent. In the ethics statement in the Methods and online submission information, please ensure that you have specified (1) whether consent was informed and (2) what type you obtained (for instance, written or verbal, and if verbal, how it was documented and witnessed). If your study included minors, state whether you obtained consent from parents or guardians. If the need for consent was waived by the ethics committee, please include this information.

If you are reporting a retrospective study of medical records or archived samples, please ensure that you have discussed whether all data were fully anonymized before you accessed them and/or whether the IRB or ethics committee waived the requirement for informed consent. If patients provided informed written consent to have data from their medical records used in research, please include this information.

Once you have amended this/these statement(s) in the Methods section of the manuscript, please add the same text to the “Ethics Statement” field of the submission form (via “Edit Submission”).

For additional information about PLOS ONE ethical requirements for human subject’s research, please refer to http://journals.plos.org/plosone/s/submission-guidelines#loc-human-subjects-research.

**The ethical approval was modified according to the editor suggestions both in Method section and in the online manuscript submission system.**

4. We suggest you thoroughly copyedit your manuscript for language usage, spelling, and grammar. If you do not know anyone who can help you do this, you may wish to consider employing a professional scientific editing service.

Whilst you may use any professional scientific editing service of your choice, PLOS has partnered with both American Journal Experts (AJE) and Editage to provide discounted services to PLOS authors. Both organizations have experience helping authors meet PLOS guidelines and can provide language editing, translation, manuscript formatting, and figure formatting to ensure your manuscript meets our submission guidelines. To take advantage of our partnership with AJE, visit the AJE website (http://learn.aje.com/plos/) for a 15% discount off AJE services. To take advantage of our partnership with Editage, visit the Editage website (www.editage.com) and enter referral code PLOSEDIT for a 15% discount off Editage services.  If the PLOS editorial team finds any language issues in text that either AJE or Editage has edited, the service provider will re-edit the text for free.

Upon resubmission, please provide the following

- The name of the colleague or the details of the professional service that edited your manuscript
- A copy of your manuscript showing your changes by either highlighting them or using track changes (uploaded as a *supporting information* file)
- A clean copy of the edited manuscript (uploaded as the new *manuscript* file)

**Thank you for this comment. The manuscript is copyedited for language usage, spelling, and grammar by an English speaking colleague Dr. Helen Appleton, Professor at the University of Oxford.**

5. Thank you for stating the following financial disclosure:

The funders had no role in study design, data collection and analysis, decision to publish, or preparation of the manuscript.

At this time, please address the following queries:

a) Please clarify the sources of funding (financial or material support) for your study. List the grants or organizations that supported your study, including funding received from your institution

b) State what role the funders took in the study. If the funders had no role in your study, please state: “The funders had no role in study design, data collection and analysis, decision to publish, or preparation of the manuscript.

c) If any authors received a salary from any of your funders, please state which authors and which funders

d) If you did not receive any funding for this study, please state: “The authors received no specific funding for this work.”

Please include your amended statements within your cover letter; we will change the online submission form on your behalf.

**The amended statements for the funders and funding source in the research work are given bellow**

**b) “The funders had no role in study design, data collection and analysis, decision to publish, or preparation of the manuscript”.**

**d) “The authors received no specific funding for this work.”**

6. Please include your tables as part of your main manuscript and remove the individual files. Please note that supplementary tables (should remain/ be uploaded) as separate "supporting information" files

**The authors are grateful to the editor for this comment. The tables and figures were included in the main manuscript and the individual files were removed according to editor suggestion.**

7. Please review your reference list to ensure that it is complete and correct. If you have cited papers that have been retracted, please include the rationale for doing so in the manuscript text, or remove these references and replace them with relevant current references. Any changes to the reference list should be mentioned in the rebuttal letter that accompanies your revised manuscript. If you need to cite a retracted article, indicate the article’s retracted status in the References list and also include a citation and full reference for the retraction notice.

**The references list was modified and rearranged in the revised manuscript according to journal references format as suggested by the editor.**

Responses to Reviewer comments

**Comments to the Author**

1. Is the manuscript technically sound, and do the data support the conclusions?

The manuscript must describe a technically sound piece of scientific research with data that supports the conclusions. Experiments must have been conducted rigorously, with appropriate controls, replication, and sample sizes. The conclusions must be drawn appropriately based on the data presented.

Reviewer #1: Yes2. Has the statistical analysis been performed appropriately and rigorously?

Reviewer #1: Yes3. Have the authors made all data underlying the findings in their manuscript fully available?

The [PLOS Data policy](http://www.plosone.org/static/policies.action#sharing) requires authors to make all data underlying the findings described in their manuscript fully available without restriction, with rare exception (please refer to the Data Availability Statement in the manuscript PDF file). The data should be provided as part of the manuscript or its supporting information, or deposited to a public repository. For example, in addition to summary statistics, the data points behind means, medians and variance measures should be available. If there are restrictions on publicly sharing data—e.g. participant privacy or use of data from a third party—those must be specified.

Reviewer #1: Yes4. Is the manuscript presented in an intelligible fashion and written in standard English?

PLOS ONE does not copyedit accepted manuscripts, so the language in submitted articles must be clear, correct, and unambiguous. Any typographical or grammatical errors should be corrected at revision, so please note any specific errors here.

Reviewer #1: Yes

5. Review Comments to the Author
Please use the space provided to explain your answers to the questions above. You may also include additional comments for the author, including concerns about dual publication, research ethics, or publication ethics. (Please upload your review as an attachment if it exceeds 20,000 characters)

Reviewer #1: Title: Seroepidemiology and Associated Risk Factors of Hepatitis B and C Virus Infections among Pregnant Women Attending Maternity Wards at Two Hospitals in Swabi, Khyber Pakhtunkhwa, Pakistan

**In the abstract:** there is no result conclusion indicated!

**We are grateful to the reviewer for this comment. The abstract of the manuscript is divided into subsections i.e. (Background & aim, Methodology, Results, Conclusion and Recommendation) and modified according to journal format. The result conclusion is also indicated according to reviewer comment and highlighted with red color.**

**Method part**

- Source population is not stated under method part

**The authors are thankful to reviewer for this comment. Source population is added in the method section under the subheading “Source and study population” and highlighted with red color.**

- Under method part: hierarchal permission process for the research was not described well and ethical principle was not stated well! (e.g. for those positive cases how the investigator manage the issue and also whether verbal or written consent was taken).

**Thanks for this comments. The hierarchal permission process for the research and ethical principle are described well in the method part and modified according to the reviewer comment.**

- Sampling techniques and sample size were not described.

**Sample techniques and sample size are stated in the method section under the subheading “Sample size and sampling” and highlighted with red color.**

- Quality assurance measures were not stated.

**In revised manuscript, the Quality assurance measures are stated in the method section under the subheading “Quality assurance measures”**

- Hospital and health center were used interchangeably (but not the same)

**The authors are grateful to the reviewer for arising this point. The authors are agreed with reviewer that Hospital and health center are not the same and should not be used interchangeably, thus the word “Hospital” is used in the entire manuscript and highlighted with red color.**

- Interpretation of the laboratory test for both viruses was not stated!

**We are grateful to the reviewer for this comment. The laboratory tests (ICT and ELISA) were described in detail in the method section under subheadings “HBsAg and HCV-Ab detection through ICT” and “HBsAg and HCV-Ab detection through ELISA”. The interpretation of the test results are stated under the theses subheadings.**

- Data analysis steps were not clear to understand how it was performed

**The authors are thankful to the reviewer for this comment. Data analysis statement was described in detail in method section under subheading “Statistical analysis” which can clearly be understood by the readers how the data was analyzed.**

**Result**

- Result part was not elaborated well (e.g by describing the socio-demographic factors except in the table)

**We are obliged to reviewer for this comment. The result section is sub-headed in “Socio demographic characteristics”. The socio-demographic characteristics are described in detail under result section which explains all the results stated in the tables.**

- Prevalence of HBV and HCV, the socio-demographic factors and associated factors shall be stated separately under different subtitles as the objective.

**The authors are agreed with the reviewer comment. Prevalence of HBV and HCV, Socio demographic characteristics and associated risk factors are stated separately under different subheadings and highlighted with red color.**

- In addition to table, if graphs are used to show the result, it will be good to easily understand the result in %.

**We appreciate the reviewer for this suggestion. In addition to tables, one graph was created for showing the prevalence results in percentages and uploaded as a separate file in the online Editorial Manager system according to reviewer comment.**

- There must be an explanation for why “Dental extraction, Receiving blood transfusion, Educational level and Residential status associated with HBsAg positivity and surgical procedure, maternal age for HCV Ab positivity”

**The authors are obliged to reviewer for this suggestion. Following the reviewer suggestions, the Dental extraction, Receiving blood transfusion, Educational level and Residential status associated with HBsAg positivity and surgical procedure, maternal age for HCV Ab positivity” are explained in result section under the subheadings “Socio demographic characteristics “, “Associated risk factors” and “Prevalence of HBV and HCV Infections” and highlighted with red color.**

**Under discussion:**

- Strength and Limitation of the study shall be specified

**We are grateful to the reviewer for this comment. Strength and limitations of the study are specified in the discussion section under the subheading “Study limitations” and highlighted with red color.**

**Under Conclusion**

- The result was not interpreted well whether it was high or low according to WHO guidelines for HBV and HCV (whether it falls in the endemic interval or hyper endemic interval and only risk factors were stated in line with the result.

**The authors appreciate the reviewer for this constructive suggestion. Some new sentences are added in the conclusion section which interpreted the results whether the prevalence of HBV and HCV is high or low according to WHO guideline.**
