# Supplementary material for: Seroepidemiology and associated risk factors of hepatitis B and C virus infections among pregnant women attending maternity wards at two hospitals in Swabi, Khyber Pakhtunkhwa, Pakistan
Source: PLoS One. 2021 Aug 20;16(8):e0255189. doi: 10.1371/journal.pone.0255189 (PMC8384242; doi:10.1371/journal.pone.0255189)
Supplement: Attachment — Submitted filename: Reviewer #1 Minor correction for the author (1).docx [file pone.0255189.s004.docx]

**Comments were incorporated by the authors but I do have the following minor comments to be considered! Suggestions were proposed in green colors as indicated below!**

**Abstract part: Methodology**

10 ml of blood was collected from each participated mother …rather put as “From a total of 375 pregnant women selected using systematic random sampling from both hospitals, 10ml of blood samples were collected…….

**Under Method parts**

**Source and study population**

All pregnant women attending maternity wards for antenatal care health facilities at Bacha Khan Medical Complex (BKMC) Shahmansoor and District Head Quarter (DHQ) Hospital Swabi from surrounding urban and rural areas of district Swabi during July 2019 -January 2020 were the source and study population: **Source population and study population is not the same**

**Source populations** were e all pregnant women attending hospital maternity wards for antenatal care at Bacha Khan Medical Complex (BKMC) Shahmansoor and District Head Quarter (DHQ) Hospital Swabi from surrounding urban and rural areas of district Swabi

**Study population** were all pregnant women attending hospital maternity wards for antenatal care at Bacha Khan Medical Complex (BKMC) Shahmansoor and District Head Quarter (DHQ) Hospital Swabi from surrounding urban and rural areas of district Swabi during July 2019 -January 2020.

Sample size and sampling ……better if it will be written as “Sample size and sampling technique/procedure”

The two hospitals were selected conveniently!

**Sample collection**

10ml of blood samples were………………….better to write as “Ten milliliter of blood samples were” starting with number is not recommended

**Ethical endorsement**

**Management of positive tested participants not addressed? (Example: those participants with positive test were given medication free of charge OR their result was communicated with respective physician for further treatment)**
