# Supplementary material for: Seroepidemiology and associated risk factors of hepatitis B and C virus infections among pregnant women attending maternity wards at two hospitals in Swabi, Khyber Pakhtunkhwa, Pakistan
Source: PLoS One. 2021 Aug 20;16(8):e0255189. doi: 10.1371/journal.pone.0255189 (PMC8384242; doi:10.1371/journal.pone.0255189)
Supplement: Attachment — Submitted filename: Responses to Reviwers.docx [file pone.0255189.s005.docx]

Dear editor,

We are grateful to you and the reviewer for suggesting some minor corrections which improved the quality of our manuscript. We are pleased to submit the revised manuscript entitled “Seroepidemiology and Associated Risk Factors of Hepatitis B and C Virus Infections among Pregnant Women Attending Maternity Wards at Two Hospitals in Swabi, Khyber Pakhtunkhwa, Pakistan” with Id PONE-D-21-11040-R1 for consideration in PLOS ONE. On the following pages, you will find our response to the reviewer comments. The reviewer comments and suggestions are highlighted with red and green color and responses to each point with normal text. On behalf of my co-authors, I thank you for your consideration of this resubmission. We appreciate your time and look forward to your response.

Sincerely,

Dr. Muhammad Israr, PhD, Postdoc (Corresponding Author)

Associate Professor, Department of Biology

The University of Haripur, KPK, Pakistan

Responses to Reviewer Comments:

**Comments were incorporated by the authors but I do have the following minor comments to be considered! Suggestions were proposed in green colors as indicated below!**

**Abstract part: Methodology** 10 ml of blood was collected from each participated mother …rather put as “From a total of 375 pregnant women selected using systematic random sampling from both hospitals, 10ml of blood samples were collected…….

Response:

We are grateful to the reviewer for this suggestion. The above suggested sentence was added in the revised version and highlighted with red color in track changes

**Under Method parts**

**Source and study population** All pregnant women attending maternity wards for antenatal care health facilities at Bacha Khan Medical Complex (BKMC) Shahmansoor and District Head Quarter (DHQ) Hospital Swabi from surrounding urban and rural areas of district Swabi during July 2019 -January 2020 were the source and study population: **Source population and study population is not the same**

**Source populations** were e all pregnant women attending hospital maternity wards for antenatal care at Bacha Khan Medical Complex (BKMC) Shahmansoor and District Head Quarter (DHQ) Hospital Swabi from surrounding urban and rural areas of district Swabi

**Study population** were all pregnant women attending hospital maternity wards for antenatal care at Bacha Khan Medical Complex (BKMC) Shahmansoor and District Head Quarter (DHQ) Hospital Swabi from surrounding urban and rural areas of district Swabi during July 2019 -January 2020.

Response:

Thanks to arise this point. Yes, we are agree with reviewer that the source population and study population are not the same and can be written in separate. In order to follow the reviewer suggestion, the source population and study population were added in method section under separate headings and highlighted with red color in revised version with track changes.

Sample size and sampling ……better if it will be written as “Sample size and sampling technique/procedure” The two hospitals were selected conveniently!

Response:

Thanks for this minor comment. The above sentence suggested by the reviewer was added in the revised version and highlighted with red color in track changes.

**Sample collection**

10ml of blood samples were………………….better to write as “Ten milliliter of blood samples were” starting with number is not recommended

Response:

The authors are grateful to the reviewer for this minor comment. The above suggested sentence was added in the revised version and highlighted with red color in track changes.

**Ethical endorsement**

**Management of positive tested participants not addressed? (Example: those participants with positive test were given medication free of charge OR their result was communicated with respective physician for further treatment)**

Response:

We are obliged to the reviewer for comment. The management of HBsAg and HCV-Ab positive tested participant was incorporated with respective physician for further treatment and addressed in the revised version under the ethical endorsement and highlighted with red color in track changes.

Note: The whole manuscript was read for any other minor mistakes and corrected accordingly.
